# Metabolomics analysis reveals both plant variety and choice of hormone treatment modulate vinca alkaloid production in *Catharanthus roseus*

**DOI:** 10.1002/pld3.267

**Published:** 2020-09-28

**Authors:** Valerie N. Fraser, Benjamin Philmus, Molly Megraw

**Affiliations:** ^1^ Molecular and Cellular Biology Program Oregon State University Corvallis OR USA; ^2^ Department of Botany and Plant Pathology Oregon State University Corvallis OR USA; ^3^ Department of Pharmaceutical Sciences Oregon State University Corvallis OR USA; ^4^ Center for Genome Research and Biocomputing Oregon State University Corvallis OR USA

**Keywords:** Catharanthus roseus, ethephon, methyl jasmonate, vinca alkaloid induction

## Abstract

The medicinal plant *Catharanthus roseus* produces numerous secondary metabolites of interest for the treatment of many diseases – most notably for the terpene indole alkaloid (TIA) vinblastine, which is used in the treatment of leukemia and Hodgkin's lymphoma. Historically, methyl jasmonate (MeJA) has been used to induce TIA production, but in the past, this has only been investigated in whole seedlings, cell culture, or hairy root culture. This study examines the effects of the phytohormones MeJA and ethylene on the induction of TIA biosynthesis and accumulation in the shoots and roots of 8‐day‐old seedlings of two varieties of *C. roseus*. Using LCMS and RT‐qPCR, we demonstrate the importance of variety selection, as we observe markedly different induction patterns of important TIA precursor compounds. Additionally, both phytohormone choice and concentration have significant effects on TIA biosynthesis. Finally, our study suggests that several early‐induction pathway steps as well as pathway‐specific genes are likely to be transcriptionally regulated. Our findings highlight the need for a complete set of'omics resources in commonly used *C. roseus* varieties and the need for caution when extrapolating results from one cultivar to another.

## 1 INTRODUCTION

**FIGURE 1:**
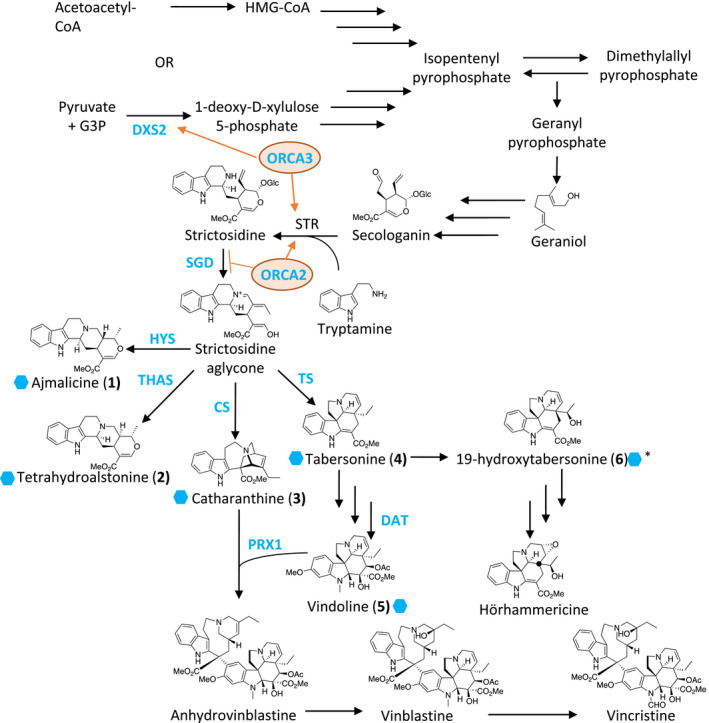
Pathway diagram from MVA and MEP to TIA. Three arrows symbolize multiple enzymatic steps and intermediates. Abbreviations in blue are the genes selected for RT‐qPCR. Orange ovals represent the TFs selected for RT‐qPCR. Compounds marked with a hexagon were quantified on LCMS. An asterisk over the hexagon denotes our hypothesis for the identity of the uncharacterized compound

Many plant‐derived secondary metabolites have chemical properties that give them therapeutic value for the treatment of cancers, hypertension, and other illnesses (Balunas & Kinghorn, 2005). In the medicinal plant *Catharanthus roseus* (L.) G. Don, the terpene indole alkaloid (TIA) family of natural products includes many valuable medicinal compounds such as the clinically used antineoplastic agents vinblastine and vincristine, as well as the antihypertensive agent ajmalicine (Figure 1). Vinblastine and vincristine, used in the treatment of lymphoblastic leukemia (Johnson, Armstrong, Gorman, & Burnett, 1963; Noble, Beer, & Cutts, 1958), are naturally produced at low levels in the leaves of the plant, which makes the chemical extraction of the two alkaloids difficult and time consuming (Tyler, 1988). Pharmaceutical scientists generally extract the more abundant precursor compounds from the leaf and perform an in vitro coupling to increase the yield of vinblastine and vincristine, which is then isolated (Ishikawa, Colby, & Boger, 2008; Potier, 1980); this process, however, can be cost prohibitive. While methyl jasmonate (MeJA) is too expensive for practical use in a large‐scale agricultural production, ethephon (a commercially available ethylene derivative) is a viable and cost‐effective option for increasing alkaloid yields prior to chemical extraction.

Over the last 50 years, laboratory studies of vinca alkaloid production has been induced *in planta* with MeJA via root uptake from growth medium or through exposure to vapor in an enclosed system (Aerts, Gisi, Carolis, De Luca, & Baumann, 1994; El‐Sayed & Verpoorte, 2004; Rijhwani & Shanks, 1998). Ethylene and its derivative ethephon (EPTN) have more recently been identified as an induction agent for the TIA pathways (Pan et al., 2010; Wang et al., 2016). Foliar application of ETPN, a compound that is quickly converted into ethylene upon uptake into the cell, does not require any special equipment and is a method that can be straightforwardly transferred from a laboratory setting into a greenhouse setting for agricultural‐scale production of these desirable compounds. If large‐scale biopharmaceutical production is the ultimate goal, foliar ETPN treatment is ideal since it is inexpensive and does not need to be reapplied to obtain the desired result.

Hairy root culture is a commonly studied system with strong potential for *C. roseus* for alkaloid production and extraction; however, it is a technically challenging system (Williams & Doran, 2000). In particular, this and similar culture systems require special equipment and impeccable sterile technique to prevent contamination. Additionally, not all precursor alkaloids of interest in the TIA pathway can be found in the roots at levels that would make extraction viable (e.g., vindoline) in the absence of further genetic engineering developments in this system (Besseau et al., 2013; Laflamme, St‐Pierre, & De Luca, 2001; O'Keefe, Mahady, Gills, Beecher, & Schilling, 1997; St‐Pierre, Vazquez‐Flota, & De Luca, 1999), and those that are present are regulated differently than in seedlings (Pan, Mustafa, Tang, Choi, & Verpoorte, 2016). Alternatively, *C. roseus* seeds are easy to germinate and are relatively fast growing in soil. Gently uprooting seedlings from the soil and thoroughly washing in deionized water allows collection of all parts of the plant in a relatively short amount of time and with minimal concern regarding contamination. These considerations make plants a good system not only for biological studies but also provides potential for greenhouse‐level scale‐up of alkaloid precursor production.

The biosynthetic pathway for terpene indole alkaloid (TIA) production in *C. roseus* has been the focus of investigation for many years starting with precursor labeling experiments (Verpoorte, van der Heijden, & Moreno, 1997) to the more recent identification of biosynthetic and regulatory genes. The TIA biosynthetic pathway begins with the coupling of the isoprene building blocks dimethylallyl pyrophosphate (DMAP) and isopentenyl pyrophosphate (IPP) to form geranyl diphosphate (GPP). Reduction in GPP to geraniol followed by a multi‐enzyme conversion results in secologanin, a common precursor for many plant natural products (Leete, 1967; Verpoorte et al., 1997). A Pictet‐Spengler reaction coupling secologanin and tryptamine (derived from decarboxylation of tryptophan by tryptophan decarboxylase [TDC]) by strictosidine reductase (STR) yields strictosidine. Deglycosylation by striosidine (SGD) followed by a spontaneous rearrangement yields strictosidine aglycone, a branch point for the biosynthesis of many TIAs including ajmalicine, tetrahydroalstonine, catharanthine, and tabersonine. Catharanthine and tabersonine are formed from strictosidine aglycone via a series of shared reactions facilitated by enzymes named precondylocarpine acetate synthase (PAS) and dihydroprecondylocarpine synthase (DPAS), followed by separate conversions by either catharathine synthase (CS) or tabersonine synthase (TS), respectively (Caputi et al., 2018). These four enzymes have also been described as geissoschizine synthase, O‐acetylstemmadenine oxidase, hydrolase 1, and hydrolase 2, respectively (Qu, Safonova, & De Luca, 2018). Tabersonine is converted in multiple steps into vindoline, which is then coupled to catharanthine by PRX1 to yield anhydrovinblastine, which is then subsequently converted into vinblastine and vincristine (Money, Wright, McCapra, Hall, & Scott, 1968; Verpoorte et al., 1997). Early steps in the TIA biosynthetic pathway are transcriptionally regulated by ORCA2 and ORCA3 (Li et al., 2013; Liu et al., 2011; Pan et al., 2012). Upregulation of ORCA2 inhibits SGD expression while upregulating STR expression (Li et al., 2013; Liu et al., 2011). ORCA3 upregulation results in the upregulation of both DXS2 (non‐mevalonate isoprenoid biosynthesis) and STR (Pan et al., 2012). Regulation of the later biosynthetic steps in the TIA pathway is currently unknown.

Many different cultivars of *C. roseus* have been developed for ornamental uses and, of these, some have also been evaluated for their utility in alkaloid production. Among these genetically diverse varieties, however, only a few have been selected for genomic and transcriptomic resource development (Góngora‐Castillo et al., 2012; Kellner et al., 2015; Pan et al., 2018; Verma, Ghangal, Sharma, Sinha, & Jain, 2014). “Little Bright Eye” (LBE) is a variety that has been commonly used for plant pathology research and was used in the initial efforts to identify the TIA biosynthetic genes. More recently, other varieties have been investigated in transcriptional and metabolomic studies (Góngora‐Castillo et al., 2012; Kellner et al., 2015; Pan et al., 2018; Verma et al., 2014). “SunStorm Apricot” (SSA) was developed for horticultural use and recently was selected for genome sequencing (Kellner et al., 2015); it remains the only sequenced *C. roseus* variety to date. Given that no single *C. roseus* variety currently has a complete set of genomic, transcriptomic, and metabolomic data available (Table S1), we wanted to investigate how alkaloid production and response to stimuli differ between the two varieties associated with the most widely used'omics resources (LBE and SSA). With this in mind, we designed a study of the alkaloid induction patterns of ethylene and MeJA in these two varieties of *C. roseus* (LBE vs. SSA). Some precursor alkaloids are restricted to certain tissues (O'Keefe et al., 1997; St‐Pierre et al., 1999); thus, we chose to perform all assays in both roots and shoots rather than in whole seedlings, which has not been addressed in previous *C. roseus* work. Additionally, testing hormonal induction in LBE has allowed us to compare observations with previous studies, while including SSA provides an opportunity for future genomic investigation into the regulation of important induction pathways.

In this work, the *in planta* effects of foliar MeJA or ETPN treatments on the metabolomic profiles in roots and shoots on alkaloid levels was investigated in both varieties. The natural differences in alkaloid levels between these varieties in roots and shoots were also investigated. Finally, this work examines the transcriptional effect of these phytohormones on the expression of genes involved in the terpene indole alkaloid biosynthetic pathway, and examines the relationship between transcriptional and metabolic profiles. We show that not only do varietal differences play a major role in alkaloid response to hormonal stimuli but also that genetic variation between SSA and LBE is substantial enough to affect wild‐type levels of alkaloids in both roots and shoots.

## 2 MATERIALS & METHODS

### 2.1 Plant material and growth

Two *Catharanthus roseus* varieties were selected for these experiments: “SunStorm Apricot” (obtained from www.expressseed.com) and “Little Bright Eyes” (obtained from www.neseeds.com). Ten to twelve seeds of a single variety were planted in 4‐inch plastic pots filled to 1 cm below the top with MetroMix potting mix (35%–45% Sphagnum moss, bark, pumice, dolomite limestone). Pots were arranged on labeled trays, which were covered with plastic domes to increase humidity until seedlings emerged through the soil. The plants were grown in an environmentally controlled growth room under a 12‐hr light/12‐hr dark photo cycle with a 22°C ambient temperature.

### 2.2 Extraction protocol validation

Prior to beginning the bulk of this study, we validated our extraction techniques to ensure that technical and biotic influences were minimized using SSA. To test our technical reproducibility, we pulverized up 10–20 shoots in liquid nitrogen and, then, after thorough mixing of the resulting powder, the powder was divided into three approximately equal portions of plant material. The replicate plant material was extracted and analyzed via LCMS (in technical duplicate) as described in the section “LCMS quantitation of *C. roseus* alkaloid”. The vindoline concentration was determined to be 12.2 ± 2.7 µg/mg wet weight. To test for biological variability, 20 plants were grown under identical conditions and randomly allocated to three samples. The samples were pulverized in liquid nitrogen, extracted with methanol, and analyzed by LCMS (in technical duplicate) as described in the section “LCMS quantitation of *C. roseus* alkaloid”. In these samples, the vindoline concentration was determined to be 10.9 ± 3.0 µg/mg wet weight. This demonstrated that our extraction protocol was reproducible and accurate.

### 2.3 Phytohormone treatments and sample collection

The “SunStorm Apricot” variety of *C. roseus* seedlings was germinated in soil and grown to 8 days post‐germination (Aerts et al., 1994; El‐Sayed & Verpoorte, 2004), at which time they were sprayed with 5 ml of DI water or 100 µM or 1 mM ethephon (dissolved in DI water). After treatment, plants were sealed inside 2‐gallon zip‐top bags and returned to the growth chamber for 24 hr. On the next day, the plants were carefully uprooted, washed with DI water, separated at the hypocotyl into roots and shoots with a surgical blade, and flash‐frozen in liquid nitrogen. Samples were stored at −80°C until they could be processed, a minimum of 24 hr.

These concentrations of ethephon were chosen as they are the manufacturer (Monterey Lawn & Garden) recommended concentration for agricultural applications (1 mM) or identical to concentration of MeJa applied (100 µM). Ethephon was mixed in DI water alone for the treatments, while the control treatment consisted of DI water. The plants were then handled as described above. Each sample from both of these experiments consisted of all the plants from a single pot; there were 12 pots for each variety. At the 1 mM concentration, the plants began showing signs of senescence, becoming yellow and wilted.

**FIGURE 2:**
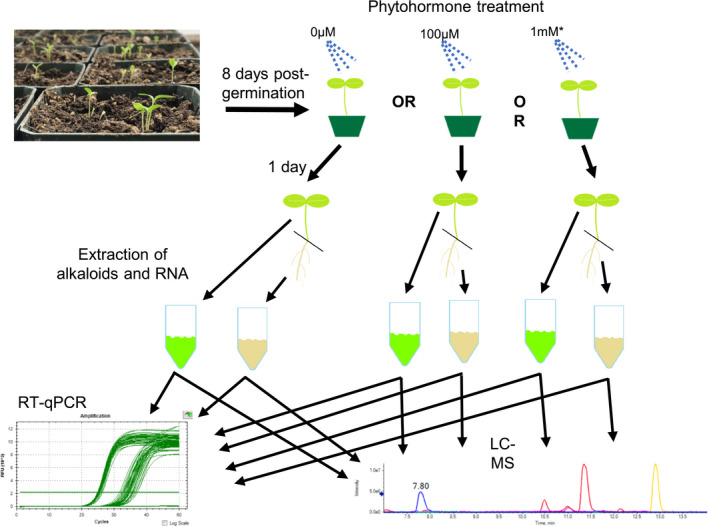
Experimental design of this study. Seeds of two Catharanthus roseus varieties (LBE and SSA) were sown and grown to 8 days post‐germination. At that time, the plants were treated with various concentrations of either ethephon or MeJA. The asterisk on the 1mM denotes that the concentration was only used for ethephon. Seedlings were harvested 24 hr after treatment, divided into roots and shoots, and flash frozen. Total alkaloid content and RNA were extracted from the frozen tissues, which were then used for LCMS and RT‐qPCR analyses

Using the data obtained from the optimization trials, we designed our larger experiment as shown in Figure 2. For this experiment, both SSA and LBE seedlings were grown to 8 days after germination. Six pots of each variety were selected at random from the trays, sprayed with a combined volume of 5 ml of DI water (ethephon control), DI water + 0.02% DMSO (methyl jasmonate control), 100 µM ethephon, 1 mM ethephon, or 100 µM methyl jasmonate + 0.02% DMSO. After treatment, the plants were handled as described above. We processed 6 replicates for each treatment. Each sample contained all of the plants from a single pot (~8 plants).

### 2.4 Alkaloid extraction

Shoots were ground in liquid nitrogen with a mortar and pestle; roots were macerated by hand with a metal spatula directly in the methanol solution to prevent sample loss during the grind and transfer process due to the small amount of tissue. Shoot extractions were performed using 1 ml methanol containing 10 µM ajmaline (internal standard) per 100 mg tissue. Root extractions were performed using 1 ml of methanol containing 1 µM ajmaline (internal standard) per 10 mg tissue. The extracts were then allowed to stand at room temperature (~22°C) for 20 min and then the cellular debris was pelleted by centrifugation (3,220× *g,* 22°C, 20 min). The cleared extracts were then filtered through a 0.22 µm nylon syringe filter to remove remaining particulate. The shoot alkaloid extracts were diluted 1:10 in methanol and 20 µl was transferred to HPLC vials containing glass sample inserts. The root extracts were used undiluted. The filled HPLC vials were stored at −80°C until they could be analyzed via LC‐MS (described below). Unfortunately, some samples were lost during processing. In the end, the final replications for each treatment were as follows: LBE E0‐S = 6; LBE E0‐R = 5; LBE E1 = 6 and 6; LBE E4 = 6 and 6; LBE M0 = 6 and 6; LBE M1 = 6 and 6; SSA E0‐S = 6; SSA E0‐R = 3; SSA E1‐S = 6; SSA E1‐R = 5; SSA E4 = 6 and 6; SSA M0‐S = 6; SSA M0‐R = 5; SSA M1 = 6 and 6.

### 2.5 LCMS quantitation of *C. roseus* alkaloids

LCMS analysis was achieved using a Shimadzu Prominence HPLC (consisting of a degasser, two LC‐10AD HPLC pumps, an autosampler, and system controller) upstream of a 3200 QTrap mass spectrometer (AbSciex). Separation was achieved using Luna C18 (2) column (150 × 2.00 mm, 3 µm) at a flow rate of 0.2 ml/ min and the following gradient, where line A was water with 0.1% (v/v) formic acid and line B was acetonitrile with 0.1% (v/v) formic acid. The column was pre‐equilibrated with 85% A/15% B. Upon injection (2 µl of prepared HPLC sample) the mobile phase composition was maintained for 1 min followed by changing the mobile phase to 60% A/40% B over 14 min using a linear gradient. The mobile phase was then changed to 0% A/100% B over the next 1 min and held at this ratio for 8 min. The mobile phase was changed to 85% A/15% B over 1 min and the column was equilibrated at 85% A/15% B for 7 min prior to the next injection. The mass spectrometer settings were as follows: MS (EMS positive mode, 50–1,500 m/z), Curtain gas, 40.0; Collision gas, Medium; IonSpray voltage, 4500.0; Temperature, 400.0; Ion Source Gas 1, 35.0; Ion Source Gas 2, 35.0; Interface heater, ON; Declustering potential, 45.0; Entrance potential, 4.0; Collision energy, 5.0, number of scans to sum, 2; scan rate, 4,000 Da/sec. MS/MS (MRM mode); for catharanthine (Q1, 337.3; Q3, 144.2; time 40 msec, CE (volts) 20.0); for tabersonine (Q1, 337.3; Q3, 305.3; time 40 msec, CE (volts) 20.0); for vinblastine (Q1, 406.2; Q3, 271.9; time 40 msec, CE (volts) 30.0); and for vincristine (Q1, 413.2; Q3, 353.4; time 40 msec, CE (volts) 30.0). Curtain gas, 40.0; Collision gas, Medium; IonSpray voltage, 4500.0; Temperature, 400.0; Ion Source Gas 1, 35.0; Ion Source Gas 2, 35.0; Interface heater, ON; Declustering potential, 45.0; Entrance potential, 10.0; Collision cell exit potential, 3.0. Data were acquired using the Analyst software package (AbSciex). LCMS grade H_2_O, acetonitrile, and methanol were purchased from MilliporeSigma. LCMS grade formic acid was purchased from Fisher Chemicals. All other chemicals were purchased from Sigma‐Aldrich and used without further purification unless otherwise specified.

Standard curves were generated by analyzing commercial standards at known concentrations using the identical LCMS settings. Vindoline, vinblastine sulfate, vincristine sulfate, and catharanthine were obtained from Cayman Chemicals. Ajmaline and tetrahydroalstonine were obtained from Extrasynthese, while ajmalicine was obtained from Millipore‐Sigma. Lochnericine and 16‐hydroxytabersonine (aka 11‐hydroxytabersonine) were obtained from MuseChem.

### 2.6 RNA extraction and qRT‐PCR

Stored tissues were ground with mortars and pestles that had been treated with RNase Zap to prevent sample degradation. The ground tissues were divided into two 2 ml microfuge tubes, which were used immediately to extract total RNA using the RNeasy Mini Kit (Qiagen) in conjunction with their RNase‐Free DNase Set (Qiagen) as directed. The total RNA for each sample was quantified on a Nanodrop (Thermo Scientific) and integrity was confirmed on a Bioanalyzer 2100 (Agilent) in the Center for Genome Research and Biocomputing Core Facilities at Oregon State University. Only samples with RINs ≥8.0 were used for two‐step qRT‐PCR. Each biological replicate was used for two technical replicates, bringing the total to four replicates for each sample. Three‐hundred nanogram of input RNA from each sample was reverse transcribed using the SuperScript RT kit (Invitrogen). qPCR and melt curve analyses were performed using the SYBR PCR kit (Qiagen) on a BioRad C1000 Touch thermocycler with a BioRad CFX96 detection system (BioRad). Transcript data were extracted using CFX Manager software (BioRad). Primers not sourced from literature were designed using PrimerQuest tool (Integrated DNA Technologies); all primers were ordered from Sigma‐Aldrich with standard desalting.

### 2.7 Data analyses

Relative intensities for each were determined from LCMS data by calculating the area under the peak (AUC) using Peakview version 2.2 (AbSciex) and then dividing that value by the AUC of our internal standard, ajmaline. Absolute concentrations were calculated from the AUC and a standard curve for each alkaloid; each quantity was then normalized using the original wet weight of the sample. We performed Welch's *t* tests to determine the significance of differences in alkaloid concentrations between varieties and two‐way ANOVA followed by Tukey pairwise comparison post hoc analyses to determine the significance of treatments. For qPCR data analysis, LinRegPCR (Ruijter et al., 2009) was used to determine primer efficiencies. Absolute copy numbers of transcripts were determined (${\bar{X}}_{0_{s}}=\Delta T*{\hat{E}}_{s}^{\left[{\bar{b}}_{a}*\log_{{\hat{E}}_{s}}\left({\bar{E}}_{a}\right)‐{\bar{C}}_{q_{s}}\right]}$) and then normalized to the absolute copy number of 40S ribosomal protein S9 (RPS9), our control gene, from the same sample. The resulting data were analyzed using ANOVA and Welch's *t* tests. All statistical analyses were performed in R (version 3.4.3). Values are considered significant below *p* = .1.

## 3 RESULTS

The terpene indole alkaloid biosynthetic pathway of *Catharanthus roseus* (TIA, Figure 1) is central to the production of its medically relevant natural products. We have designed a large study to examine the transcriptional regulation of vinca alkaloid production in the roots and shoots of seedlings from two varieties that are of interest to medicinal chemistry and genomics researchers (Figure 2). Here, we present our findings.

Alkaloid levels substantially differ between varieties. A comparison of the spectrometric results of the untreated control plants highlights notable differences between the plant varieties themselves. As these control plants were only sprayed with deionized water and the pots were arranged randomly to avoid positional effects, changes observed are attributable to variety. Vinblastine and vincristine were below the limit of detection of our LCMS system and, thus, are not discussed here. Additionally, we were unable to separate ajmalicine and tetrahydroalstonine despite multiple attempts, as they have identical masses, fragmentation patterns, and retention times. Therefore, we report these two compounds here as a single value relative to the internal standard.

**FIGURE 3:**
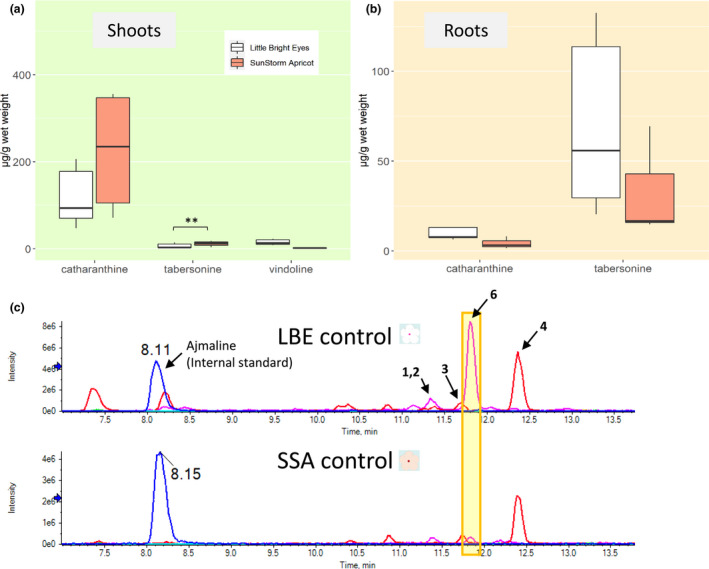
Alkaloid concentrations differ greatly between untreated plants of the two varieties. **Denotes a *p*‐value ≤ .01 (a) In shoots, SunStorm Apricot has a higher concentration of catharanthine and tabersonine, while Little Bright Eye has a much greater concentration of vindoline. (b) In roots, Little Bright Eye has higher concentrations of catharanthine, tabersonine, and ajmalicine/tetrahydroalstonine. (c) Representative LCMS traces from the 0 µM ethephon treatment group; even in the control treatment, there are obvious differences between the varieties. The white flower represents LBE, and the peach flower represents SSA. Labeled peaks are as follows: 1 = ajmalicine; 2 = tetrahydroalstonine; 3 = catharanthine; 4 = tabersonine; 6 = 19‐hydroxytabersonine (putative assignment)

In shoots, untreated SSA plants have markedly higher levels of tabersonine (Welch's *t* test, *p* ≤ .01), while LBE has a higher concentration of vindoline (Figure 3a). The mean vindoline concentration is greater in LBE than in SSA, but the difference is not significant due to LBE having much more intra‐varietal variation (Welch's *t* test, *p* = .1033). In roots, untreated LBE has higher concentrations of catharanthine and tabersonine, but not statistically significant (Welch's *t* test, *p* = .13 and 0.2, respectively) (Figure 3b). An alkaloid with a mass to charge ratio of 353 is present at high levels in the roots of untreated LBE and at lower levels in the roots of untreated SSA (Figure 3c). Overall, we observe that important differences arise in alkaloid concentration between varieties and between the tissues of these varieties, even without the application of an induction agent.

Induction of alkaloid levels differs markedly based on which phytohormone is used. In both shoots and roots, both methyl jasmonate (MeJA) and ethephon (ETPN) either caused an increase in alkaloid level or had no effect; there was no evidence of a significant decrease in any of the alkaloids examined.

**FIGURE 4:**
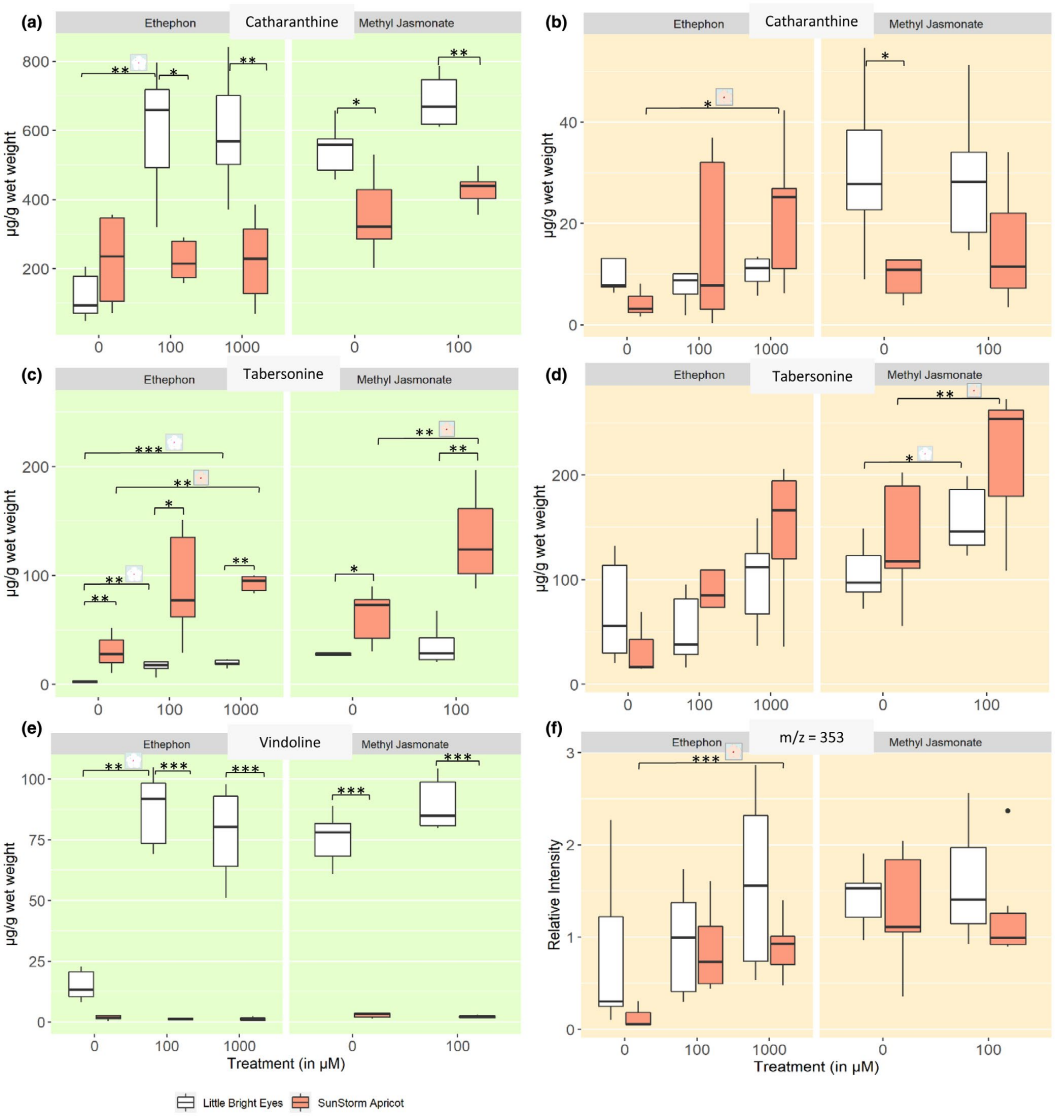
Alkaloid concentrations differ between varieties and treatment. *Denotes a *p*‐value ≤ .05; **denotes a *p*‐value ≤ .01; ***denotes a *p*‐value ≤ .001; all represented statistics are from Welch's *t* test post hoc analyses. Significance markers with a white flower represent treatment differences in LBE, while those with a peach flower represent treatment differences in SSA. (a) Catharanthine concentrations increased in LBE shoots after treatment with both hormones, but only with methyl jasmonate in SSA shoots. (b) In roots, catharanthine increased markedly in SSA after treatment with ethephon. (c) In shoots, tabersonine levels increase greatly in SSA upon treatment with either phytohormone, but only after treatment with ethephon in LBE. (d) Tabersonine levels increase significantly in the roots of both varieties after treatment with phytohormone. (e) Vindoline concentration increases significantly in LBE shoots after treatment with ethephon. (f) The amount of the unidentified alkaloid present relative to the internal standard increased significantly in the roots of SSA after treatment with ethephon

For catharanthine and the combined ajmalicine/tetrahydroalstonine peak, treatment with MeJA increased the concentration in the shoots of both varieties but not significantly (Figure 4, Figure S1). Application of MeJA significantly increased the concentration of tabersonine in SSA, but not in LBE (Figure 4; Welch's *t* test, *p* ≤ .01). For vindoline, there was a small increase in LBE, which was not significant, and no increase in SSA.

Treatment of LBE with ETPN increased the concentration of catharanthine, tabersonine, and vindoline at both concentrations (Figure 4a,c,e). For the ajmalicine/tetrahydroalstonine peak, a small increase in the mean was observed that was not statistically significant. In SSA shoots, ETPN only significantly increases the levels of tabersonine at both treatment concentrations (Figure 4c). None of the other alkaloids examined showed increases in concentration.

In the roots of LBE, MeJA treatment only increased the concentration of tabersonine (Figure 4, and Figure S1). For SSA, MeJA treatment significantly increased the concentration of tabersonine (Figure 4); the mean amount of ajmalicine/tetrahydroalstonine increased, although the increase was not significant (Figure S1). The mean concentration of catharanthine was not significantly changed by MeJA in the roots of either variety.

Treatment of LBE with ethephon did not significantly alter the concentrations of any of the alkaloids examined in this study in the roots. In the case of SSA, treatment with 1 mM ethephon significantly increased concentrations of catharanthine, ajmalicine/tetrahydroalstonine, and an unidentified alkaloid at m/z = 353 in the roots (vida infra; Figure 4 and Figure S1). Treatment of SSA with ethephon increases tabersonine four‐fold in roots, but cannot be considered statistically significant (Figure 4d).

Overall, ethephon significantly increased the levels of a catharanthine, tabersonine, and vindoline in LBE shoots while MeJA did not significantly increase the amount of any alkaloid examined. In the SSA shoot samples only tab was significantly increased in both the ETPN and MeJA treatments. All of the alkaloids mentioned in this study are precursors in the TIA pathway. Additionally, the interaction between treatment and variety was significant for some of the alkaloids. In shoots, catharanthine and vindoline were increased significantly by the interaction of ETPN and variety while the interaction of MeJA and variety significantly affected tabersonine. In the roots, however, none of the alkaloids had significant interaction effects. This latter result may be due to the foliar application of the phytohormones.

**FIGURE 5:**
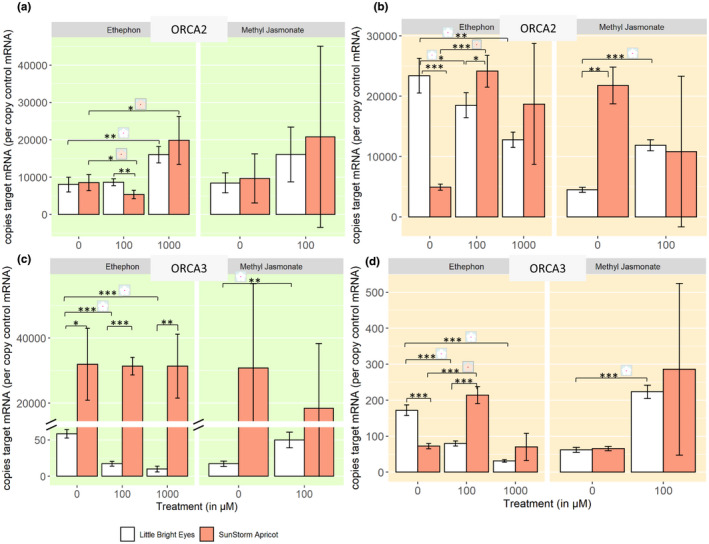
Expression of key regulatory genes is transcriptionally regulated upon phytohormone treatment. *Denotes a *p*‐value ≤ .05; **denotes a *p*‐value ≤ .01; ***denotes a *p*‐value ≤ .001; all represented statistics are from Welch's *t* test post hoc analyses. Significance markers with a white flower represent treatment differences in LBE, while those with a peach flower represent treatment differences in SSA. (a) ORCA2 transcripts in shoots. (b) ORCA2 transcripts in roots. (c) ORCA3 transcripts in shoots (+zoomed in panel). (d) ORCA3 transcripts in roots

Master regulators are upregulated by hormonal induction. In the shoots of both varieties, the higher concentration of ETPN induces an increase in the number of ORCA2 transcripts (Figure 5a; ANOVA, *p* ≤ .001). ORCA2 transcripts in roots, however, respond oppositely in the two varieties: increasing with ETPN treatment in SSA while decreasing in LBE and decreasing with MeJA treatment in SSA increasing in LBE (Figure 5b; ANOVA, *p* ≤ .001). In untreated plants, ORCA3 transcripts are present at significantly higher levels in the shoots of SSA than in the same tissue in LBE (Figure 5c; Welch's *t* test, *p* ≤ .001). These levels in SSA, however, do not respond to treatment with ETPN – unlike in LBE, where they are significantly decreased (ANOVA, *p* ≤ .01). In roots, treatment with MeJA increases transcripts in both varieties; however, we see opposing effects depending on variety in shoots (Figure 5d). The changes in ORCA3 in SSA do mirror the changes seen in catharanthine and ajmalicine in roots, although this is not true of ORCA3 in LBE. Treatment with phytohormones does have an effect on the expression of these master regulators, although both the basal level of expression and the induction effect appears to vary between the two varieties.

Evidence supports transcriptional regulation of key pathway steps. We found two key primary metabolic enzymes in upstream pathways change upon phytohormone treatment. In the mevalonate‐independent pathway (MEP), the DXS2 transcript level increased in the roots of our young plants treated with MeJA (Figure S2c); in the mevalonate (MVA) meanwhile, HMGS transcript abundance decreased in the shoots of the MeJA‐treated plants (Figure S2a).

**FIGURE 6:**
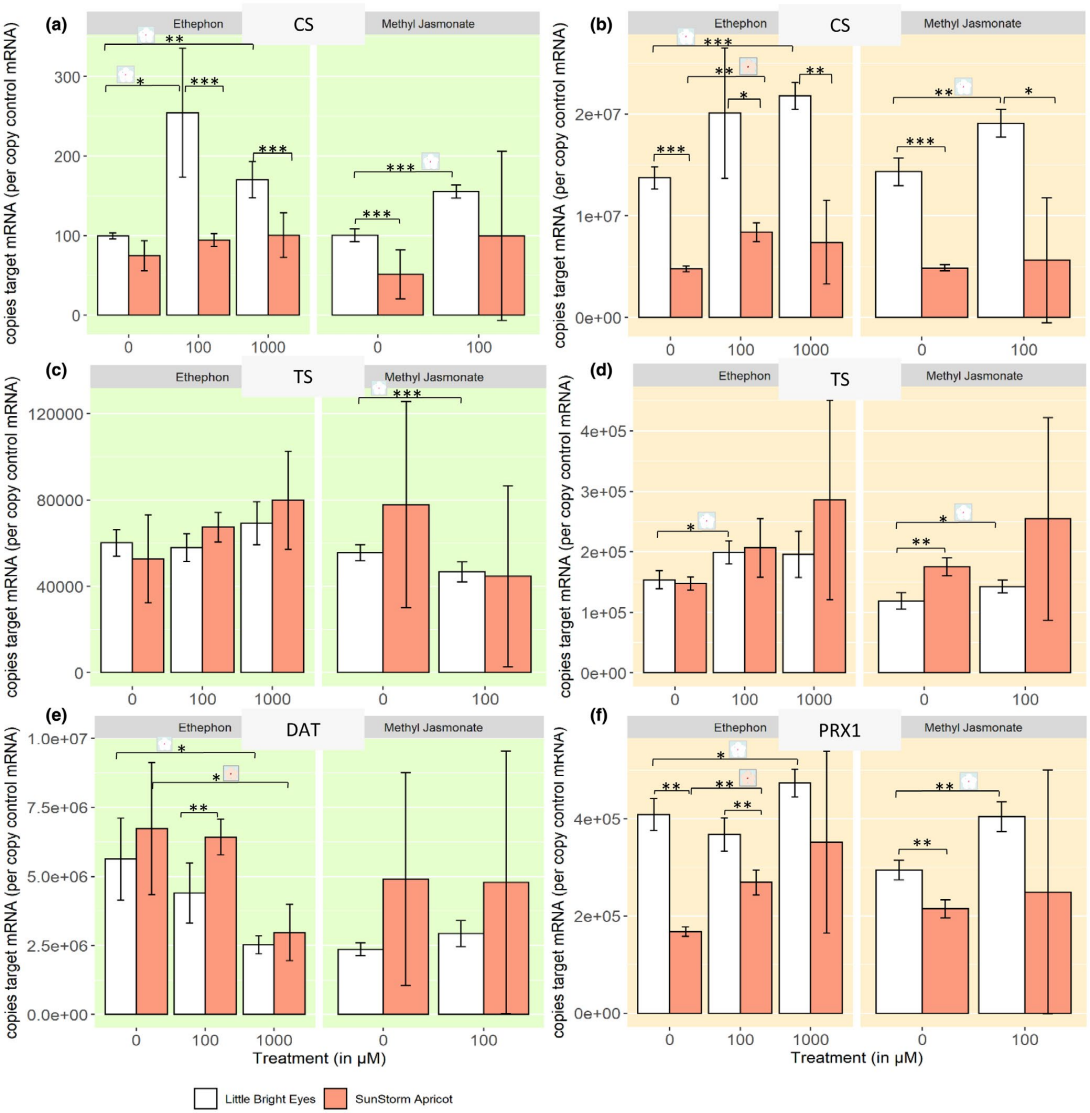
Expression of some key enzymes in the TIA pathway is transcriptionally regulated upon phytohormone treatment. *Denotes a *p*‐value ≤ .05; **denotes a *p*‐value ≤ .01; ***denotes a *p*‐value ≤ .001; all represented statistics are from Welch's *t* test post hoc analyses. Significance markers with a white flower represent treatment differences in LBE, while those with a peach flower represent treatment differences in SSA. (a) CS shoots. (b) CS roots. (c) TS shoots. (d) TS roots. (e) DAT. (f) PRX1 transcript levels in roots increase after treatment with ethephon in both varieties and decrease after treatment with MeJA, but only in LBE

In shoots, the catharanthine synthase (CS) transcript levels increase in all groups except for ETPN‐treated SSA, which is consistent with the trends we observed in catharanthine production (Figure 6a; ANOVA, *p* ≤ .001). While catharanthine appears to be transcriptionally regulated in LBE roots by both ETPN and MeJA, CS levels in SSA roots are only increased by ETPN (Figure 6b: ANOVA, *p* ≤ .001). In the shoots of both varieties, tabersonine concentrations do not appear to be transcriptionally regulated, as tabersonine synthase (TS) transcript levels increase after treatment with ETPN and decrease after treatment with MeJA, which is not at all consistent with the trends in alkaloid concentration (Figure 6c). In roots, on the other hand, tabersonine does appear to be transcriptionally regulated in both varieties, as a significant induction of TS transcripts after treatment with ETPN is observed which are correlated with observed changes in the tabersonine concentrations (Figure 6d).

Interestingly, although vindoline concentrations increase significantly in LBE shoots upon treatment with ETPN, there is no associated increase in deacetylvindoline *O*‐acetyltransferase (DAT) mRNA levels; in fact, we observe a significant decrease in both varieties (Figure 6e; ANOVA, *p* ≤ .01). Shoots treated with MeJA, however, do have similar increases in DAT mRNA and vindoline (Welch's *t* test, *p* ≤ .1).

In roots and shoots of both of the varieties, ETPN treatment caused a statistically significant increase in the transcription of PRX1 (Figure 6f, Figure S3; ANOVA, *p* ≤ .001). Meanwhile, MeJA decreased transcription levels in all tissues of SSA and, significantly, in the roots of LBE (Figure 6f, Figure S3; Welch's *t* test, *p* ≤ .01). The change in ajmalicine/tetrahydroalstonine is consistent with the patterns that we observed in SSA roots and the increase that we saw in SGD transcripts is seen in all SSA tissues (Figure S3; ANOVA, *p* ≤ .001).

## 4 DISCUSSION

Previous studies have investigated the genomics or metabolomics of different *C. roseus* varieties (Chung et al., 2011; Kim et al., 2007; Magnotta, Murata, Chen, & De Luca, 2006). While many of the examined varieties in these works are used mainly for ornamental purposes, several are used medicinally. None of the studies, however, include both LBE and SSA – which would allow for the utilization of available genomic and transcriptomic resources to inform future bioengineering efforts (Table S1). We selected these two varieties for this reason.

Questions generated by our study. One of the compounds that exhibited a clear concentration difference between LBE and SSA was the uncharacterized alkaloid (compound 6). Due to this alkaloid's mass‐to‐charge ratio (*m/z* = 353) and its retention time with respect to the other identified peaks, we suspect that this compound is a hydroxylated tabersonine. We endeavored to confirm our hypothesis about our uncharacterized alkaloid's identity using commercially available standards for alkaloids with the appropriate molecular weight (including 11‐hydroxytabersonine, yohimbine, and lochnericine); however, none of the commercially available standards exhibited the same retention time as this peak. We, therefore, posit that this compound is 19‐hydroxytabersonine, which is only present in *C. roseus* roots (Shanks, Bhadra, Morgan, Rijhwani, & Vani, 1998) and for which we were unable to identify a commercial supplier or create our own standard. If this is, in fact, 19‐hydroxytabersonine, a significant increase is interesting, although not necessarily a desirable, result as it channels tabersonine to hörhammericine, echitovenine, or minovincine – none of which are clinically used (Shanks et al., 1998).

Similarly to observations in previous studies (Aerts et al., 1994; El‐Sayed & Verpoorte, 2004; Jaleel, Gopi, Gomathinayagam, & Panneerselvam, 2009; Pan et al., 2010; Wang et al., 2016; Zhang et al., 2018), treatment of both LBE and SSA seedlings with either methyl jasmonate or ethephon induced the production of various precursor alkaloids. These hormones also affected the expression levels of several key biosynthetic enzymes and transcription factors. Previous studies concluded that ethylene induces the MVA pathway while jasmonate induces the MEP in older seedlings (Pan et al., 2018; Zhang et al., 2018). It is interesting to note that our results for key enzymes from these pathways (DXS2 in MEP and HMGS in MVA) in roots and shoots treated through foliar application of the phytohormones show different induction patterns than were observed in Pan et al. (2018) and Zhang et al. (2018), where treatments were applied to entire seedlings via hydroponic supplementation. A different physiological outcome in roots and shoots may be expected given that the immediate uptake happens through a different tissue; in some aspects, however, our results are not directly comparable, as outcome specific to the individual plant parts was not investigated in these past studies. Regardless, both DXS2 and HMGS are key enzymes in the formation of the indole component of terpene indole alkaloids, so a change in transcript abundance due to phytohormone treatment could have downstream effects on concentration of each alkaloid.

Examining varietal differences in the roles of master regulators. The ORCA family of transcription factors has been documented as central regulators of early‐stage TIA intermediate production in *C. roseus* (Li et al., 2013; Liu et al., 2011; Pan et al., 2012). Previous studies in hairy root culture have shown that the overexpression of ORCA2 significantly increases concentrations of catharanthine and vindoline levels while decreasing tabersonine levels (Li et al., 2013; Liu et al., 2011), but our results do not appear to have the same correlations. These results underscore the need for broader investigation in different varieties and the care that must be taken when extrapolating results from one variety of *C. roseus* to inform results or pathway engineering of another variety. As ORCA3 positively regulates two key genes in the TIA pathway (Pan et al., 2012), the significant difference between its expression in SSA and LBE makes its promoter an interesting target for further investigation and future bioengineering efforts. Our study also supports ORCA2 as a candidate for engineering, as its transcript levels increased upon treatment. As with any master regulator, however, there is the possibility of the generation of off‐target effects such as activation of potential repressors, so a careful investigation into genes controlled by these two TFs would be necessary.

Linking transcriptional changes to metabolite production. Additionally, we selected seven biosynthetic genes that encode key enzymes directly related to the biosynthesis of terpene indole alkaloids. Of these genes, five perform important reactions in the path toward vinblastine; the remaining two genes are involved in reactions that branch off from the vinblastine biosynthesis pathway but catalyze the formation of other medicinally relevant alkaloids (e.g., reserpine, etc.). Given the evidence from past studies that there are important pathway differences between roots and shoots, we felt that it was necessary to investigate the expression of these genes in these separate plant organs, as this information will be useful for engineering alkaloid production in biopharmaceutical settings. The observed results are intriguing.

Catharanthine synthase (CS) and tabersonine synthase (TS) produce catharanthine and tabersonine, respectively, and were recently determined to be two of the four missing enzymes in the TIA pathway (Caputi et al., 2018; Qu et al., 2018). We were particularly interested in discovering how the various phytohormone treatments affected their transcription, since relatively little research has been published on these genes since their discovery in the last two years. In shoots, the changes in the concentration of catharanthine are consistent with the observed changes in CS transcript number, suggesting that this particular step in the TIA pathway is transcriptionally regulated; this does not, however, appear to be the case in roots. Tabersonine production, meanwhile, does not appear to be transcriptionally regulated, as observed changes in TS transcript levels do not correspond to the changes in the alkaloid concentrations. One explanation for this behavior could be that an enzyme directly upstream acts as a bottleneck in the pathway, while the amount of TS present remains consistent because it is expressed at a level that is sufficient to handle an increased amount of substrate. Alternatively, post‐transcriptional or translational changes caused by the hormone treatments could be responsible for the observed increases in tabersonine concentration.

The changes in vindoline and the associated biosynthetic enzyme DAT are puzzling. SSA shoots treated with MeJA have similar increases in DAT mRNA and vindoline (Welch's *t* test, *p* ≤ .1), which is consistent with the changes in vindoline concentration observed in previous studies of *C. roseus* plants overexpressing DAT (Wang et al., 2012). In SSA, shoots treated with ethephon, however, the concentration of the alkaloid increased dramatically upon induction, even though the number of DAT transcripts decreases. Perhaps the ethephon caused a post‐translational modification that increased the efficiency of the enzyme (Chen & Bleecker, 1995). Further investigation into this response is needed. Although α‐3’,4’‐anhydrovinblastine and vinblastine levels were below the detection limit of our mass spectrometer, they are still key alkaloids, which is why we chose to examine PRX1. Previous work in cell culture demonstrated a correlation among the overexpression of PRX1, an increase in the number of SGD transcripts, and an increase in ajmalicine accumulation (Jaggi, Kumar, & Sinha, 2011). The results observed for these genes and for the ajmalicine/tetrahydroalstonine peak in SSA are consistent with these patterns. Re‐examination of the alkaloid extracts with a higher‐sensitivity mass spectrometer would allow us to examine the changes in α‐3′,4′‐anhydrovinblastine, vinblastine, and vincristine concentrations caused by the hormone treatments and how they relate to the increases observed in PRX1.

Overall, production of many key TIAs appears to be transcriptionally regulated in at least one tissue. ETPN and MeJA induce approximately equal numbers of the biosynthetic genes that code for key enzymes in the TIA pathway. They also induce genes upstream of the TIA pathway, which may be useful information for future bioengineering attempts. When taken in conjunction with the changes observed in alkaloid concentrations and with consideration of the cost of large‐scale application, ETPN appears to be a viable option with considerable potential for alkaloid production in a biopharmaceutical setting.

## 5 CONCLUSIONS

In summary, our work demonstrates that choice of *C. roseus* variety, phytohormone type, and treatment concentration all have an impact on the levels of key alkaloids in each plant organ. Either a genomic or transcriptomic resource is available for the two varieties investigated here, but neither variety has both. The differing baseline metabolic profile as well as the differing responses to phytohormone treatment emphasize the importance of choosing an appropriate variety for one's desired outcomes. Additionally, optimization of treatments is crucial; timing of phytohormone application and harvest, as well as the concentration applied, can have significant effects on both the health of the plants and the induced changes in alkaloid concentrations. Finally, this study suggests that ethephon is a viable and agriculturally relevant induction agent for key alkaloids in a large‐scale biopharmaceutical production setting.

## CONFLICT OF INTEREST

The authors declare no conflict of interest associated with the work described in this manuscript.

## AUTHOR CONTRIBUTIONS

BP and MM conceived the project and secured funding for the work. VF, BP, and MM designed the experiments. VF and BP conducted the experiments and analyzed the data. VF, BP, and MM wrote the manuscript. All authors have given approval to the final version of the manuscript.

6

**FIGURE 7:**
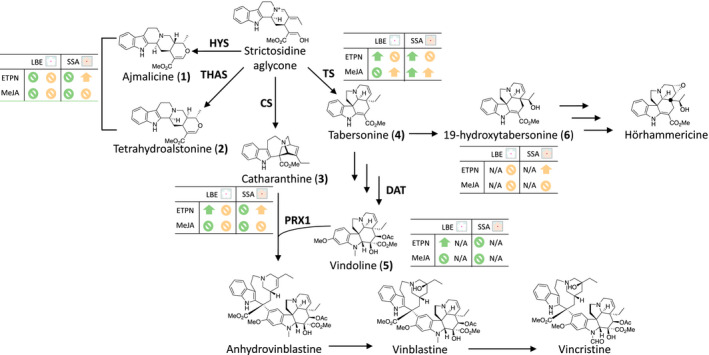
Summary of changes in metabolite levels in response to phytohormone treatment. Colored arrows pointing up reflect an increase in either concentration or peak intensity relative to ajmaline (internal standard). A symbol reflects no change. The color of the arrow or symbol reflects the tissue: green = shoot, tan = root

## Supporting information

Fig S1

Fig S2

Fig S3

Table S1

Table S2

Table S3

Table S4

Table S5

Table S6

Table S7

Table S8

Table S9
