# Supplementary material for: Metabolomics analysis reveals both plant variety and choice of hormone treatment modulate vinca alkaloid production in Catharanthus roseus
Source: Plant Direct. 2020 Sep 28;4(9):e00267. doi: 10.1002/pld3.267 (PMC7520646; doi:10.1002/pld3.267)
Supplement: Supplementary file 1 — Fig S1 [file PLD3-4-e00267-s001.pdf]

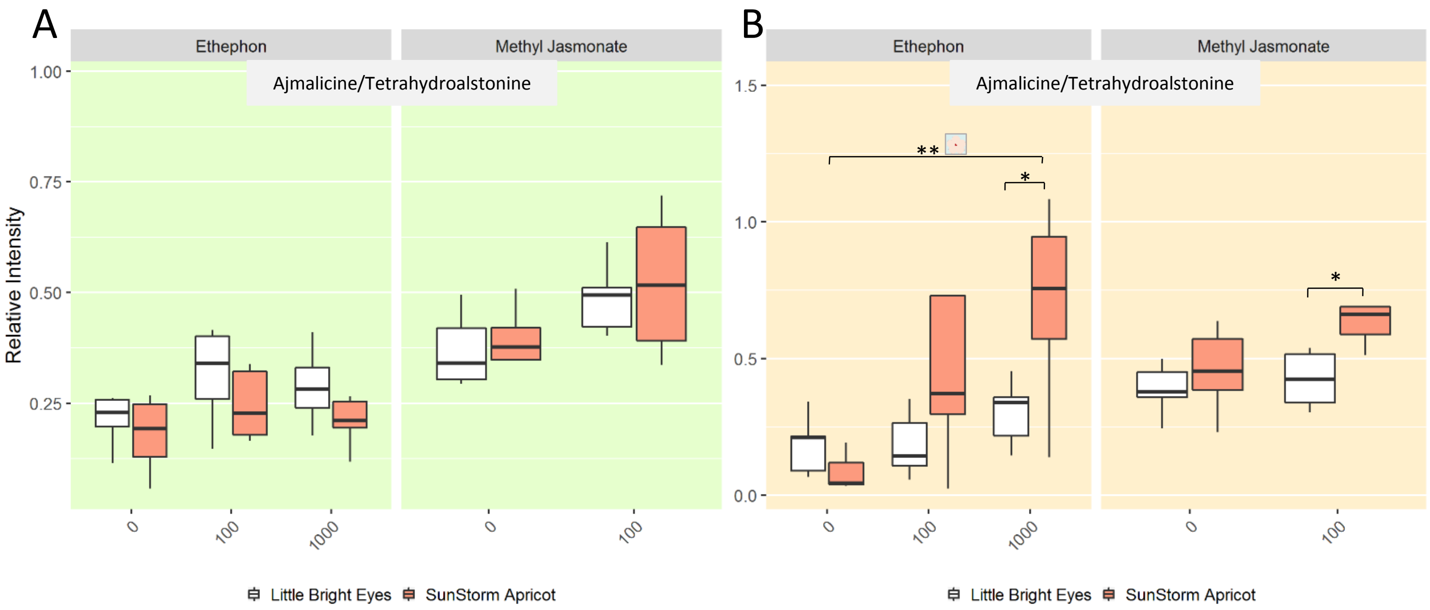

Figure S1. The concentration of ajmalicine/tetrahydroalstonine relative to our internal standard (ajmaline) appears to increase upon hormone treatment. * denotes a p-value ≤ 0.05; ** denotes a p-value ≤ 0.01; all represented statistics are from Welch's t-test post-hoc analyses. Significance markers with a white flower represent treatment differences in LBE, while those with a peach flower represent treatment differences in SSA.(A) Peak intensity relative to internal standard in shoots; (B) peak intensity relative to internal standard in roots.
