# Supplementary material for: Metabolomics analysis reveals both plant variety and choice of hormone treatment modulate vinca alkaloid production in Catharanthus roseus
Source: Plant Direct. 2020 Sep 28;4(9):e00267. doi: 10.1002/pld3.267 (PMC7520646; doi:10.1002/pld3.267)
Supplement: Supplementary file 2 — Fig S2 [file PLD3-4-e00267-s002.pdf]

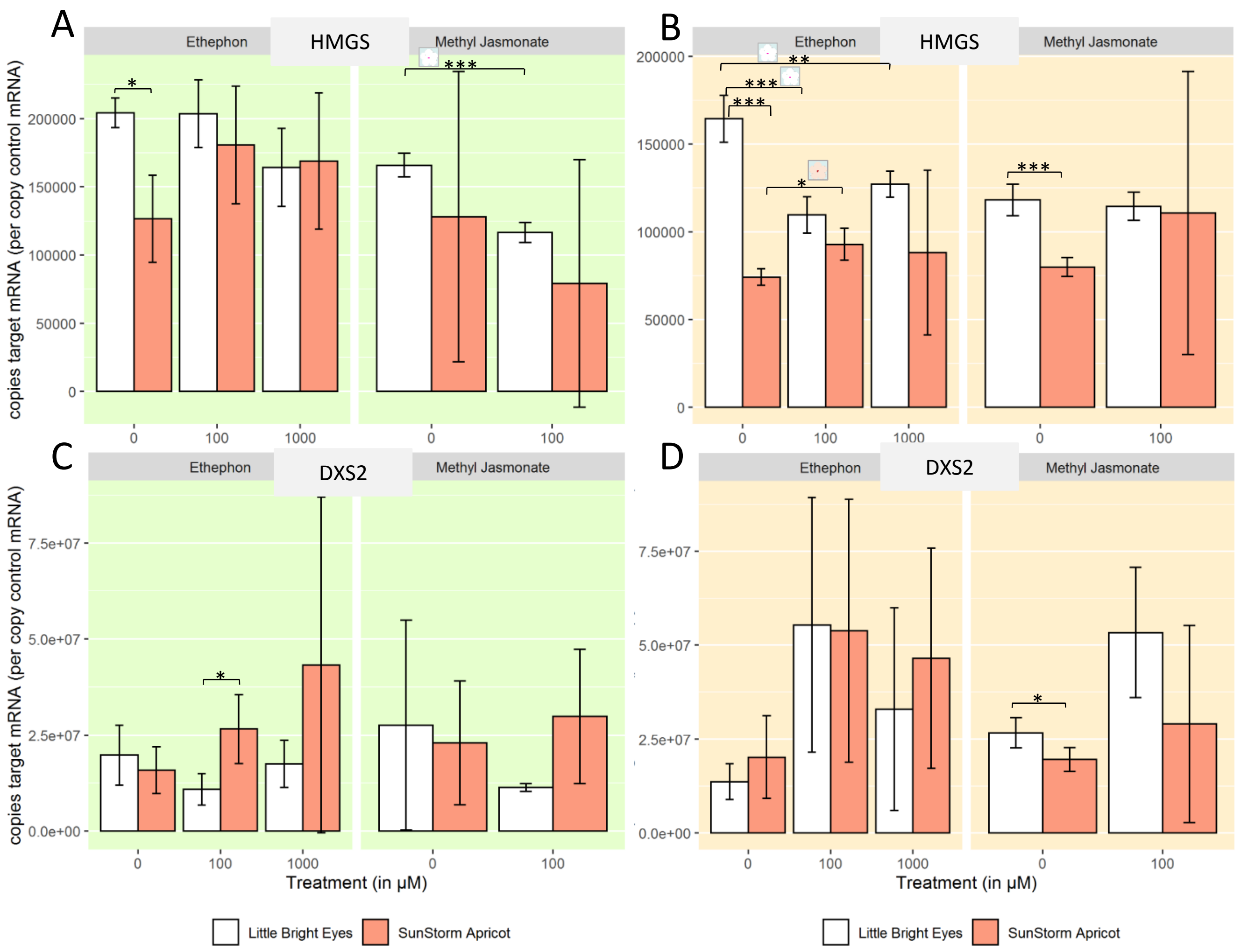

Figure S2. Expression of genes from pathways upstream of the TIA pathway appear to be transcriptionally regulated by hormone treatment. * denotes a p-value ≤ 0.05; ** denotes a p-value ≤ 0.01; *** denotes a p-value ≤ 0.001; all represented statistics are from Welch's t-test post-hoc analyses. Significance markers with a white flower represent treatment differences in LBE, while those with a peach flower represent treatment differences in SSA. (A) HMGS/MVA pathway in shoots (B) HMGS/MVA pathway in roots (C) DXS2/MEP pathway in shoots (D) DXS2/MEP pathway in roots.
