# Supplementary material for: Metabolomics analysis reveals both plant variety and choice of hormone treatment modulate vinca alkaloid production in Catharanthus roseus
Source: Plant Direct. 2020 Sep 28;4(9):e00267. doi: 10.1002/pld3.267 (PMC7520646; doi:10.1002/pld3.267)
Supplement: Supplementary file 3 — Fig S3 [file PLD3-4-e00267-s003.pdf]

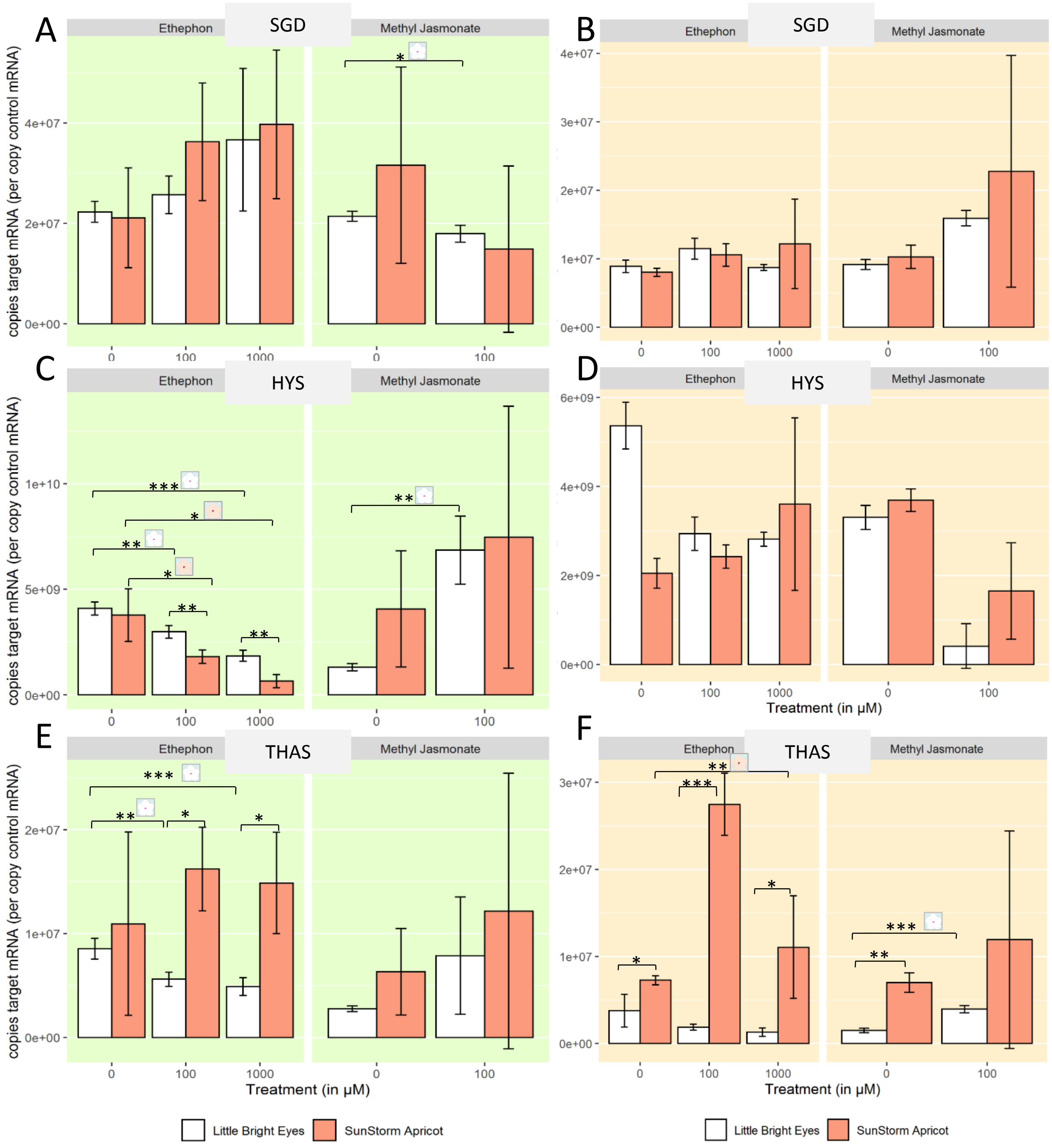
A
SGD
Ethephon
Methyl Jasmonate
B
SGD
Ethephon
Methyl Jasmonate
C
HYS
Ethephon
Methyl Jasmonate
D
HYS
Ethephon
Methyl Jasmonate
E
THAS
Ethephon
Methyl Jasmonate
F
THAS
Ethephon
Methyl Jasmonate
copies target mRNA (per copy control mRNA)
Treatment (in μM)
0
100
1000
Little Bright Eyes
SunStorm Apricot

Figure S3. Expression of some key enzymes in the TIA pathway are transcriptionally regulated upon phytohormone treatment. * denotes a p-value ≤ 0.05; ** denotes a p-value ≤ 0.01; *** denotes a p-value ≤ 0.001; all represented statistics are from Welch's t-test post-hoc analyses. Significance markers with a white flower represent treatment differences in LBE, while those with a peach flower represent treatment differences in SSA. (A) SGD expression in shoots (B) SGD expression in roots. (C) HYS expression in shoots (D) HYS expression in roots (E) THAS expression in shoots (F) THAS expression in roots.
