# Supplementary material for: Metabolomics analysis reveals both plant variety and choice of hormone treatment modulate vinca alkaloid production in Catharanthus roseus
Source: Plant Direct. 2020 Sep 28;4(9):e00267. doi: 10.1002/pld3.267 (PMC7520646; doi:10.1002/pld3.267)
Supplement: Supplementary file 4 — Table S1 [file PLD3-4-e00267-s004.pdf]

Variety	Genome	Transcriptome	Metabolome	Source
SunStorm Apricot	Yes	No	Yes	Kellner 2015; Chung 2011; Magnotta 2006
Little Bright Eye	No	Yes	No	Góngora-Castillo 2012
Prabal	No	Yes	No	Verma 2014
63 other varieties	No	No	Yes	Chung 2011
49 other varieties	No	No	Yes	Magnotta 2006

Table S1. The ’omics data available for selected varieties of *Catharanthus roseus.*
