# Supplementary material for: Metabolomics analysis reveals both plant variety and choice of hormone treatment modulate vinca alkaloid production in Catharanthus roseus
Source: Plant Direct. 2020 Sep 28;4(9):e00267. doi: 10.1002/pld3.267 (PMC7520646; doi:10.1002/pld3.267)
Supplement: Supplementary file 5 — Table S2 [file PLD3-4-e00267-s005.pdf]

	Little Bright Eye					SunStorm Apricot				
(µg/g wet wt)	0 µM ETPN	100 µM ETPN	1 mM ETPN	0 µM MeJA	100 µM MeJA	0 µM ETPN	100 µM ETPN	1 mM ETPN	0 µM MeJA	100 µM MeJA
Tabersonine										
Shoots	3.64 ± 3.77	18.22 ± 8.55	21.35 ± 6.77	29.73 ± 7.91	35.65 ± 18.29	30.03 ± 15.77	108.66 ± 83.40	94.38 ± 31.16	63.02 ± 25.02	133.40 ± 43.37
Roots	70.40 ± 53.35	69.93 ± 72.42	100.07 ± 46.39	105.26 ± 28.84	156.99 ± 33.52	33.59 ± 26.85	108.57 ± 98.05	147.04 ± 64.93	135.07 ± 77.26	217.91 ± 67.9
Catharanthine										
Shoots	174.45 ± 189.09	601.37 ± 181.58	595.06 ± 171.18	545.68 ± 75.48	637.64 ± 165.61	224.03 ± 134.52	287.11 ± 196.48	224.77 ± 125.45	352.43 ± 121.26	429.60 ± 50.29
Roots	13.28 ± 10.93	11.57 ± 11.48	17.26 ± 18.26	30.38 ± 15.88	28.91 ± 13.69	4.30 ± 3.19	16.02 ± 16.68	22.13 ± 13.78	11.85 ± 8.96	15.33 ± 11.84
Vindoline										
Shoots	25.03 ± 28.64	81.39 ± 27.24	77.51 ± 19.08	75.66 ± 10.55	81.60 ± 26.18	1.76 ± 0.99	1.68 ± 1.15	1.26 ± 0.76	2.82 ± 1.08	2.21 ± 0.53

Table S2. Mean alkaloid concentations detected (shown ± standard deviation) in each tissue under each treatment condition.
