# Supplementary material for: Metabolomics analysis reveals both plant variety and choice of hormone treatment modulate vinca alkaloid production in Catharanthus roseus
Source: Plant Direct. 2020 Sep 28;4(9):e00267. doi: 10.1002/pld3.267 (PMC7520646; doi:10.1002/pld3.267)
Supplement: Supplementary file 6 — Table S3 [file PLD3-4-e00267-s006.pdf]

ANOVAs

All treatments:

Shoots:

	Treatment	Variety	Interaction
**Tetrahydroalstonine**	5.35e-05 ***	0.483	0.730
**Ajmalicine**	0.00155 **	0.39483	0.79135
**Catharanthine (MRM)**	2.67e-05 ***	1.83e-06 ***	0.0128 *
**Tabersonine (MRM)**	0.000234 ***	1.31e-09 ***	0.032702 *
**Vindoline**	0.00026 ***	< 2e-16 ***	0.00029 ***

Signif. codes: 0 '***'| 0.01 '**'| 0.05 '*' | 0.1 '.'

Roots:

	Treatment	Variety	Interaction
**Tetrahydroalstonine**	0.009913 **	0.000816 ***	0.096387 .
**Ajmalicine**	0.11075	0.00126 **	0.16752
**Catharanthine (MRM)**	0.203	0.108	0.162
**Tabersonine (MRM)**	0.000198 ***	0.048320 *	0.495856

Signif. codes: 0 '***'| 0.01 '**'| 0.05 '*' | 0.1 '.'

Table S3. p-values for absolute concentrations of alkaloids from ANOVA.
