# Supplementary material for: Metabolomics analysis reveals both plant variety and choice of hormone treatment modulate vinca alkaloid production in Catharanthus roseus
Source: Plant Direct. 2020 Sep 28;4(9):e00267. doi: 10.1002/pld3.267 (PMC7520646; doi:10.1002/pld3.267)
Supplement: Supplementary file 7 — Table S4 [file PLD3-4-e00267-s007.pdf]

A

Variety comparisons

Shoots:

	Eth 0µM (control)	Eth 100µM	Eth 1mM	MeJA 0µM (control)	MeJA 100µM
**Catharanthine**	0.6133	0.01656 *	0.001983 **	0.009982 **	0.02625 *
**Tabersonine**	0.008407 **	0.0449 *	0.001858 **	0.02097 *	0.001604 **
**Vindoline**	0.1033	0.000813 ***	0.0001867 ***	1.143e-05 ***	0.0006946 ***

Roots:

	Eth 0µM (control)	Eth 100µM	Eth 1mM	MeJA 0µM (control)	MeJA 100µM
**Catharanthine**	0.1388	0.6362	0.6143	0.03932 *	0.0954 .
**Tabersonine**	0.2476	0.4871	0.1831	0.3548	0.08791 .

B

Treatment effects

Shoots:

		Catharanthine	Tabersonine	Vindoline
**LBE**	Eth (0:100)	0.002572 **	0.006743 **	0.00582 **
	Eth (0:1000)	0.002411 **	0.0005549 ***	0.004941 **
	MeJA (0:100)	0.2558	0.4912	0.623
**SSA**	Eth (0:100)	0.5329	0.06897 .	0.893
	Eth (0:1000)	0.9923	0.002391 **	0.3502
	MeJA (0:100)	0.1951	0.008793 **	0.2516

Roots

		Catharanthine	Tabersonine
**LBE**	Eth (0:100)	0.8036	0.9903
	Eth (0:1000)	0.6636	0.341
	MeJA (0:100)	0.8765	0.01715 *
**SSA**	Eth (0:100)	0.2052	0.1716
	Eth (0:1000)	0.0239 *	0.009398 **
	MeJA (0:100)	0.5842	0.06147 .

Table S4. p-values for absolute concentrations of alkaloids analyses from Welch's t-test pairwise comparisons post-hoc. * denotes a p-value ≤ 0.05; ** denotes a p-value ≤ 0.01; *** denotes a p-value ≤ 0.001; (A) p-values for pairwise comparisons between varieties; (B) p-values for pairwise comparisons of treatments for each variety.
