# Supplementary material for: Metabolomics analysis reveals both plant variety and choice of hormone treatment modulate vinca alkaloid production in Catharanthus roseus
Source: Plant Direct. 2020 Sep 28;4(9):e00267. doi: 10.1002/pld3.267 (PMC7520646; doi:10.1002/pld3.267)
Supplement: Supplementary file 8 — Table S5 [file PLD3-4-e00267-s008.pdf]

A Variety comparisons

Shoots:

	Eth 0µM (control)	Eth 100µM	Eth 1mM	MeJA 0µM (control)	MeJA 100µ<br>100µM
**Ajmalicine/ Tetrahydroalstonine**	0.2879	0.8004	0.318	0.8737	0.4245
**Catharanthine**	0.0491 *	0.0111 *	0.003289 **	0.0105 *	0.0341 *
**Tabersonine**	0.0252 *	0.03674 *	0.002858 **	0.02719 *	0.001498 **
**Vindoline**	0.00638 **	4.117e-5 ***	1.997e-6 ***	8.102e-8***	2.591e-5 ***

Roots:

	Eth 0µM (control)	Eth 100µM	Eth 1mM	MeJA 0µM (control)	MeJA 100µM
**Ajmalicine/ Tetrahydroalstonine**	0.2417	0.1926	0.03341 *	0.4341	0.01938 *
**Unknown 353**	0.169	0.8337	0.1536	0.6517	0.3356
**Catharanthine**	0.2881	0.6361	0.616	0.04065 *	0.1079
**Tabersonine**	0.1592	0.4952	0.1926	0.385	0.07137 .

B Treatment effects

Shoots:

		Ajmalicine/ Tetrahydroalstonine	Catharanthine	Tabersonine	Vindoline
**LBE**	Eth (0:100)	0.2536	0.1021	0.08171 .	0.4615
	Eth (0:1000)	0.4328	0.1353	0.01179 *	0.3351
	MeJA (0:100)	0.3265	0.353	0.5384	0.9569
**SSA**	Eth (0:100)	0.2455	0.4596	0.05323 .	0.97
	Eth (0:1000)	0.3365	0.7701	0.003449 **	0.5153
	MeJA (0:100)	0.08547 .	0.194	0.008402 **	0.2849

Roots

		Ajmalicine/ Tetrahydroalstonine	Catharanthine	Tabersonine	Uncharacterized m/z = 353
**LBE**	Eth (0:100)	0.9717	0.8488	0.9638	0.7975
	Eth (0:1000)	0.1226	0.617	0.2827	0.2184
	MeJA (0:100)	0.5281	0.953	0.01491 *	0.6319
**SSA**	Eth (0:100)	0.1265	0.2159	0.1933	0.06465 .
	Eth (0:1000)	0.005827 **	0.02762 *	0.01057 *	0.001765 **
	MeJA (0:100)	0.05296 .	0.5081	0.04527 *	0.9346

Table S5. p-values for peak intensity of alkaloids relative to internal standard (ajmaline) from Welch's t-test pairwise comparisons post-hoc. * denotes a p-value ≤ 0.05; ** denotes a p-value ≤ 0.01; *** denotes a p-value ≤ 0.001; red boxes are around the uncharacterized m/z = 353 peak. (A) p-values for pairwise comparisons between varieties; (B) p-values for pairwise comparisons of treatments for each variety.
