# Supplementary material for: Metabolomics analysis reveals both plant variety and choice of hormone treatment modulate vinca alkaloid production in Catharanthus roseus
Source: Plant Direct. 2020 Sep 28;4(9):e00267. doi: 10.1002/pld3.267 (PMC7520646; doi:10.1002/pld3.267)
Supplement: Supplementary file 9 — Table S6 [file PLD3-4-e00267-s009.pdf]

qPCR stats (normalized)

Shoots:

	Treatment	Variety	Interaction
**SGD**	1.09e-06 ***	0.5798	0.0305 *
**CS**	1.32e-07 ***	3.52e-12 ***	0.00152 **
**TS**	1.71e-13 ***	0.00928 **	1.73e-06 ***
**HYS**	5.18e-09 ***	0.0017 **	3.84e-08 ***
**THAS**	0.028701 *	0.000266 ***	0.006317 **
**DAT**	0.00329 **	0.18643	0.01432 *
**ORCA2**	2.73e-08 ***	0.0763 .	0.7814
**ORCA3**	0.0130 *	4.44e-11 ***	0.0378 *
**PRX1**	0.000301 ***	0.465309	0.014337 *
**DXS2**	0.122	0.623	0.518
**HGMS**	5.26e-05 ***	2.80e-06 ***	0.232

Signif. codes: 0 ‘***’| 0.01 ‘**’| 0.05 ‘*’ | 0.1 ‘.’

Roots:

	Treatment	Variety	Interaction
**SGD**	2.57e-06 ***	0.00299 **	0.00122 **
**CS**	5.49e-08 ***	1.05e-12 ***	0.00225 **
**TS**	1.98e-10 ***	3.02e-07 ***	1.21e-05 ***
**HYS**	< 2e-16 ***	0.0662 .	4.62e-16 ***
**THAS**	4.93e-10 ***	3.64e-15 ***	3.39e-10 ***
**ORCA2**	7.42e-12 ***	1.30e-05 ***	9.96e-13 ***
**ORCA3**	5.08e-08 ***	0.005728 **	0.000266 ***
**PRX1**	1.54e-11 ***	2.86e-08 ***	0.00062 ***
**DXS2**	0.00164 **	0.88019	0.26284
**HGMS**	1.52e-09 ***	3.79e-10 ***	0.000998 ***

Signif. codes: 0 ‘***’| 0.01 ‘**’| 0.05 ‘*’ | 0.1 ‘.’

Table S6. p-values for normalized RT-qPCR from ANOVA.
