# Supplementary material for: Metabolomics analysis reveals both plant variety and choice of hormone treatment modulate vinca alkaloid production in Catharanthus roseus
Source: Plant Direct. 2020 Sep 28;4(9):e00267. doi: 10.1002/pld3.267 (PMC7520646; doi:10.1002/pld3.267)
Supplement: Supplementary file 10 — Table S7 [file PLD3-4-e00267-s010.pdf]

Shoots (at 95% confidence):

	SGD	CS	TS	HYS	THAS
**LBE**					
E0 vs E1	0.1828	0.0312 *	0.6502	0.00217 **	0.0041 **
E0 vs E4	0.1355	0.0076 **	0.1804	3.777e-05 ***	0.0016 **
M0 vs M1	0.0166 *	7.517e-05 ***	0.0260 *	0.00601 **	0.1682
**SSA**					
E0 vs E1	0.0976 .	0.1282	0.2501	0.0473 *	0.3360
E0 vs E4	0.0885 .	0.1895	0.1257	0.0125 *	0.4746
M0 vs M1	0.2403	0.4377	0.3751	0.3716	0.4516

	DAT	ORCA2	ORCA3	PRX1	DXS2	HMGS
**LBE**						
E0 vs E1	0.2345	0.5960	7.656e-05 ***	0.2316	0.1039	0.9682
E0 vs E4	0.0221 *	0.0017 **	2.460e-05 ***	0.7947	0.6560	0.0625 .
M0 vs M1	0.0863 .	0.1268	0.0059 **	0.0116 *	0.3203	1.380e-04 ***
**SSA**						
E0 vs E1	0.8151	0.0484 *	0.9285	0.4976	0.1209	0.0944 .
E0 vs E4	0.0433 *	0.0330 *	0.9417	0.0125 *	0.3004	0.2115
M0 vs M1	0.9716	0.4330	0.4768	0.7450	0.5855	0.5112

Varieties

	Eth 0µM (control)	Eth 100µM	Eth 1mM	MeJA 0µM (control)	MeJA 100µM
**SGD**	0.8267	0.1690	0.7763	0.3730	0.7357
**CS**	0.0772 .	0.0282 *	0.0093 **	0.0454 *	0.3712
**TS**	0.5309	0.0917 .	0.4365	0.4203	0.9399
**HYS**	0.6547	0.0017 **	0.0013 **	0.1385	0.8606
**THAS**	0.6229	0.0120 *	0.0246 *	0.1875	0.5828
**DAT**	0.4679	0.0252 *	0.4589	0.2783	0.4920
**ORCA2**	0.7190	0.0045 **	0.3258	0.7555	0.7308
**ORCA3**	0.0102 *	0.00018 ***	0.00784 **	0.0975 .	0.1610
**PRX1**	0.0493 *	0.149645	0.0727 .	0.9298	0.9335
**DXS2**	0.4954	0.0309 *	0.3236	0.7836	0.1249
**HMGS**	0.01204 *	0.4017	0.8788	0.5297	0.4735

Table S7. p-values for normalized RT-qPCR in shoots from Welch’s t-test pairwise comparisons post-hoc.
