# Supplementary material for: Metabolomics analysis reveals both plant variety and choice of hormone treatment modulate vinca alkaloid production in Catharanthus roseus
Source: Plant Direct. 2020 Sep 28;4(9):e00267. doi: 10.1002/pld3.267 (PMC7520646; doi:10.1002/pld3.267)
Supplement: Supplementary file 11 — Table S8 [file PLD3-4-e00267-s011.pdf]

Roots (at 95% confidence):
Treatment

	SGD	CS	TS	THAS	HYS
**LBE**					
E0 vs E1	0.0352 *	0.1404	0.0102 *	0.1318	0.00044 ***
E0 vs E4	0.7443	1.0464e-04 ***	0.1158	0.0736 .	0.00128 **
M0 vs M1	0.00014 ***	0.00255 **	0.03599 *	0.00013 ***	0.00025 ***
**SSA**					
E0 vs E1	0.0482 *	0.0026 **	0.0908 .	0.0012 **	0.1311
E0 vs E4	0.2969	0.2932	0.1919	0.2899	0.2070
M0 vs M1	0.2366	0.8262	0.4141	0.4890	0.0294 *

	ORCA2	ORCA3	PRX1	DXS2	HMGS
**LBE**					
E0 vs E1	0.0361 *	0.00021 ***	0.1349	0.0893 .	0.00078 ***
E0 vs E4	0.0022 **	0.00016 ***	0.0248 *	0.2474	0.00528 **
M0 vs M1	8.820e-05 ***	0.00012 ***	0.0016 **	0.0509 .	0.5620
**SSA**					
E0 vs E1	0.00051 ***	0.00062 ***	0.00219 **	0.1488	0.0180 *
E0 vs E4	0.0706 .	0.9302	0.1439	0.1717	0.5944
M0 vs M1	0.1769	0.1621	0.8028	0.5235	0.5005

Variety

	Eth 0µM (control)	Eth 100µM	Eth 1mM	MeJA 0µM (control)	MeJA 100µM
**SGD**	0.1741	0.4535	0.3710	0.3053	0.4782
**CS**	0.000278 ***	0.03403 *	0.0037 **	0.00052 ***	0.0195 *
**TS**	0.5280	0.7979	0.3571	0.0014 **	0.2743
**HYS**	0.00011 ***	0.0698 .	0.4782	0.0835 .	0.1028
**THAS**	0.02918 *	0.00067 ***	0.0447 *	0.00156 **	0.2910
**ORCA2**	0.00079 ***	0.0166 *	0.3216	0.0013 **	0.8774
**ORCA3**	0.00016 ***	0.00073 ***	0.1297	0.4201	0.6366
**PRX1**	0.00033 ***	0.00460 **	0.2854	0.0012 **	0.3041
**DXS2**	0.334845	0.95096	0.5236	0.0344 *	0.1805
**HMGS**	0.000305 ***	0.0507 .	0.19485	0.000798 ***	0.9302

Table S8. p-values for normalized RT-qPCR in roots from Welch’s t-test pairwise comparisons post-hoc.
