# Supplementary material for: Metabolomics analysis reveals both plant variety and choice of hormone treatment modulate vinca alkaloid production in Catharanthus roseus
Source: Plant Direct. 2020 Sep 28;4(9):e00267. doi: 10.1002/pld3.267 (PMC7520646; doi:10.1002/pld3.267)
Supplement: Supplementary file 12 — Table S9 [file PLD3-4-e00267-s012.pdf]

Gene	Forward primer	Reverse primer	Genbank Accession	Product length
strictosidine β-D-glucosidase (SGD)	GGAGGATCTGCTTATCAGTGTG	TGGCTGGATATCGGTTTGT	AF112888.1	91nt
catharanthine synthase (CS)	CTCCTGGCGGGATGAATAAC	GGAAACCAGGGTAACCAACA	MF770512	139nt
tabersonine synthase (TS)	AGATGCTCCTGGTGGAAATG	CAACCATGGAAATCAGCAACC	MF770513	104nt
heteroyohimbine synthase (HYS)	AGCAATCAGATTTGCCAAGG	GGGTTACTGTTGAGCAAGAAAG	KU865325.1	120nt
class III peroxidase 1 (PRX1)	TTCCATTGGGAAGAAGAGATGG	TTAGGAGGGCACTTGTGTTG	AM236087.1	96nt
tetrahydroalstonine synthase (THAS)	TTTAGGTGCACCAGAAATGC	TTCCTTCATACTCCCAGCAG	KM524258.1	97nt
6-17-O-acetylvindoline O-acetyltransferase (DAT)	AGAGACCTAGTCCTTCCCAAAC	AAAGCAACCGCCAAACCT	AF053307.1	101nt
ORCA2	CGTTTCAACTCCGTGGTTCTA	ATCGGCGTCTAGGACTTACT	AJ238740.1	92nt
ORCA3	CCAGCTCGGAATTGACTTCTAC	GGCTACCGGGTTTCTGTATTT	EU072424.1	96nt
1-deoxy-D-xylulose-5-phosphate synthase (DSX2)	CGAATGGGGTTTTAATGAGG	GAGTGGAGAAATGGGAGGAA	DQ848672.1	61nt
hydroxymethylglutaryl-CoA synthase (HMGS)	CTCAATGAGTATGACGGCAGTT	AGACGACCAATTTGCTTTGG	JF739871.1	72nt
RPS9 (reference gene)	TCAGTTTCTACCGGAACTATGG	GCTTCAACTCTGCATCCAATC	AJ749993	84nt

Table S9: qPCR primers; CS and TS primer sequences were obtained from Caputi *et al,* 2018. DSX2 and HMGS primer sequences were obtained from Zhang *et al,* 2012.
